# Population Genomics Training for the Next Generation of Conservation Geneticists: ConGen 2018 Workshop

**DOI:** 10.1093/jhered/esaa001

**Published:** 2020-03-10

**Authors:** Amanda Stahlke, Donavan Bell, Tashi Dhendup, Brooke Kern, Samuel Pannoni, Zachary Robinson, Jeffrey Strait, Seth Smith, Brian K Hand, Paul A Hohenlohe, Gordon Luikart

**Affiliations:** 1 Institute for Bioinformatics and Evolutionary Studies, University of Idaho, Moscow, ID; 2 Wildlife Biology Program, College of Forestry and Conservation, University of Montana, Missoula, MT; 3 Department of Forest and Park Services, Ugyen Wangchuck Institute for Conservation and Environmental Research, Bumthang, Bhutan; 4 Division of Biological Sciences, College of Humanities and Sciences, University of Montana, Missoula, MT; 5 Department of Plant and Microbial Biology, University of Minnesota, St. Paul, MN; 6 Flathead Lake Biological Station, Division of Biological Sciences, College of Humanities and Sciences, University of Montana, Missoula, MT; 7 Department of Fisheries and Wildlife, Michigan State University, East Lansing, MI

**Keywords:** adaptive capacity, conservation genetics pedagogy, effective population size, evolutionary significant units, population genomic data analysis

## Abstract

The increasing availability and complexity of next-generation sequencing (NGS) data sets make ongoing training an essential component of conservation and population genetics research. A workshop entitled “ConGen 2018” was recently held to train researchers in conceptual and practical aspects of NGS data production and analysis for conservation and ecological applications. Sixteen instructors provided helpful lectures, discussions, and hands-on exercises regarding how to plan, produce, and analyze data for many important research questions. Lecture topics ranged from understanding probabilistic (e.g., Bayesian) genotype calling to the detection of local adaptation signatures from genomic, transcriptomic, and epigenomic data. We report on progress in addressing central questions of conservation genomics, advances in NGS data analysis, the potential for genomic tools to assess adaptive capacity, and strategies for training the next generation of conservation genomicists.

Informing conservation efforts is one of the most important and challenging needs of the genomic era (Allendorf 2017; Lewin *et al.* 2018; Hunter *et al.* 2018). To help meet this challenge, 16 experts from many areas of genomic data analysis met to discuss and teach recent analytical approaches at the 10th International Population Genetics Data Analysis Workshop for Conservation (“ConGen”), held at Flathead Biological Station in September of 2018. The goal of the workshop was to train participants to apply rigorous theory and novel molecular and computational approaches in conservation and population genetics.

Since the first ConGen in 2006 (https://cibio.up.pt/congen/index.html), the molecular and computational tools accessible to conservation have grown in number and matured (Andrews and Luikart 2014; Benestan *et al.* 2015; Hendricks *et al.* 2018). ConGen 2018 participants originated from 16 countries and had a wide range of research questions and career stages including undergraduate and graduate (Masters and PhD) students, postdoctoral scholars, university faculty, laboratory technicians, and governmental agency scientists. This diversity of origins and perspectives enriched the questions, comments, discussions, and overall learning experience.

Historically, ConGen and other conservation genetics courses have focused mainly on questions that require and use only ~10–20 well-tested markers (e.g., microsatellites) such as hybridization, inbreeding, population structure, and loss of genetic diversity (Allendorf 2017). Today, the variety of molecular tools, amount of genetic data, and range of computational approaches have greatly expanded. Conservation genomics can be broadly defined as the application of genome-wide markers and new technologies to address problems in conservation. A more narrow-sense definition requires high-density loci to characterize locus- or gene-specific patterns and address conceptually novel questions that were intractable using traditional approaches (Allendorf *et al.* 2010; Garner *et al.* 2016; Allendorf 2017; Luikart *et al.* 2018).

Throughout this genetics-to-genomics transition, many authors, including those of previous ConGen workshop reviews, have reflected on this paradigm shift. They have noted the best practices for data production and quality control (filtering), experimental design, computational approaches, career guidance, and the increasing roles of women (Andrews and Luikart 2014; Benestan *et al.* 2015; Shafer *et al.* 2017; Hendricks *et al.* 2018). In this meeting review of ConGen 2018, we focus our reflection on training the next generation of researchers in conservation genomics through the novel components of this year’s workshop: progress in understanding central concepts including assessing population differentiation and conservation units, estimation of effective population size, molecular data production and analysis for diverse empirical systems (Figure 1), and prospects for understanding genomic vulnerability.

**Figure 1.**
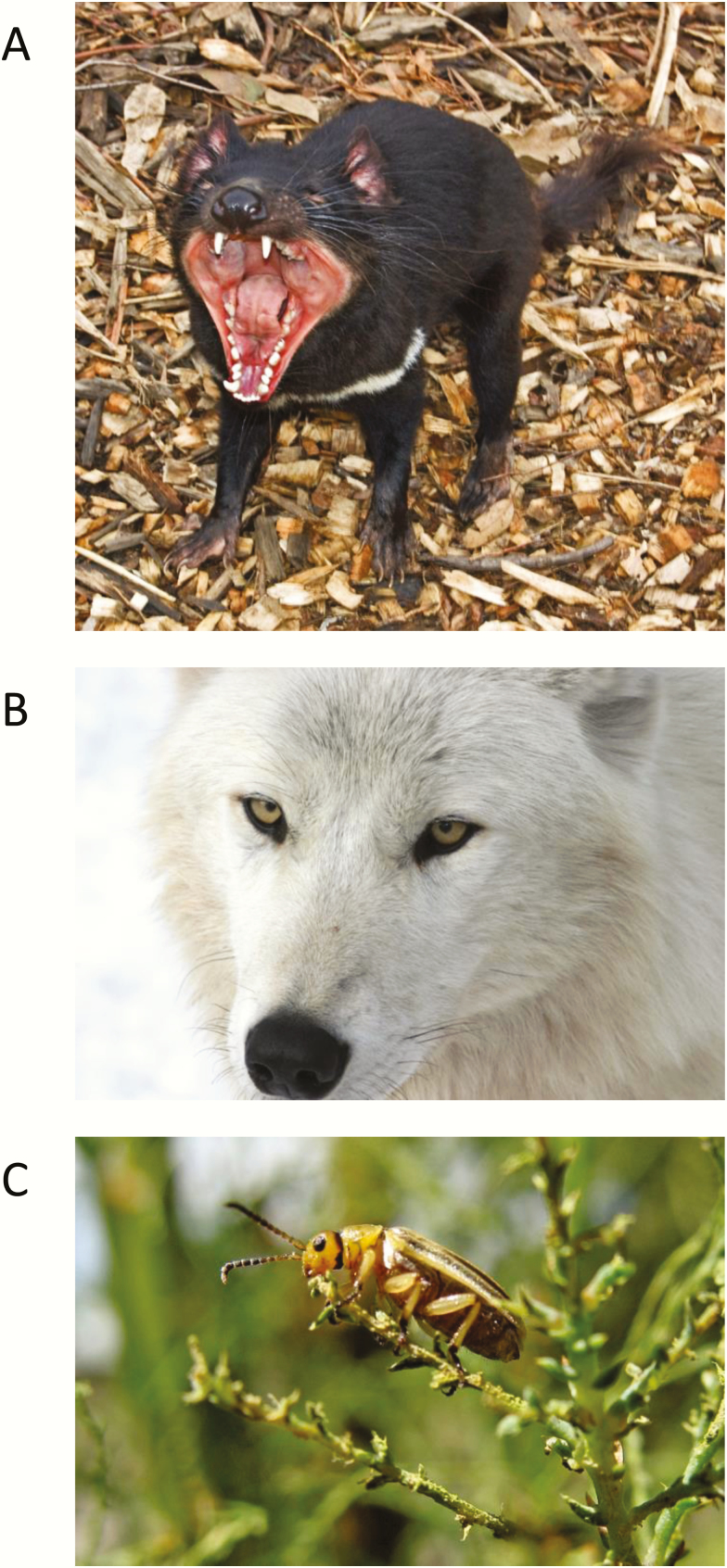
Empirical examples provided by instructors at ConGen 2018 across a broad range of data types, questions, and taxa. (**A**) RAD-Capture and GWAS in characterizing the genetic architecture of disease-related traits in Tasmanian devils (*Sarcophilus harisii*; Margres *et al.* 2018), (**B**) targeted-capture, demographic modeling, and linkage-disequilibrium analysis in understanding the evolutionary history of color polymorphism of the gray wolf (*Canis lupus*; Schweizer *et al*. 2018), and (**C**) RADseq and analysis of population structure in identifying range expansion and hybridization of the tamarisk beetle (*Diorhabda* spp.), a recently introduced biocontrol agent (Bean and Dudley 2018). Photographs by (A) Menna Jones, (B) Marco Musiani, and (C) Ed Kosmicki, respectively, reproduced with permission. See online version for full colors.

## Progress in Central Concepts

### Populations, ESUs, and CUs: How Do You Identify Them Using Genomics?

Defining biologically meaningful management units within species is challenging (Waples and Gaggiotti 2006; Bradshaw *et al*. 2018; Waples and Lindley 2018). For conservation, an Evolutionarily Significant Unit (ESU) is a distinct population or group of populations that can be protected under the U.S. Endangered Species Act (ESA; USFWS and NMFS 1996; Waples and Lindley 2018). In Robin Waples’ (Northwest Fisheries Science Center) lecture on ESUs, he explained that while there is no single or universal definition of a population, the competing definitions of ESUs emphasize 2 criteria: 1) substantial reproductive isolation and 2) an important component of the evolutionary legacy of the species (Waples 1991; Waples and Gaggiotti 2006). Evolutionary legacy refers to having distinct or different adaptations probably important for species persistence. Molecular genetic data have long been used to assess the isolation criterion for identifying ESUs, but prior to the age of genomics, the evolutionary significance of a population was difficult to determine and was largely inferred by ecological observations.

With genomic data, we can now identify loci, alleles, and surrounding chromosomal regions associated with adaptive differentiation, which improves our capacity to define ESUs while taking into account both demographic and selective processes (Funk *et al.* 2012, 2018). Incorporating adaptive variation into ESU listing raises theoretical and practical challenges (Funk *et al.* 2018). Mike Miller’s ConGen 2018 lecture on an early-migration phenotype in salmonids demonstrated this challenge, wherein previous studies found little evidence for genetic isolation, but locus-specific analysis and simulation modeling provided strong evidence for this phenotype as an important component of the species’ evolutionary legacy (Box 1).

Box 1. How will an adaptive locus influence listing of distinct salmonid populations under the Endangered Species Act (ESA) of the United States? Chinook salmon (*Oncorhynchus tshawytscha*) and steelhead (*O. mykiss*) have distinct spring (premature) and fall (mature, normal) migratory phenotypes (called runs) in several river basins across western United States. The spring-run phenotype differs substantially in behavior and physiology but has declined in abundance throughout the ranges of both species. Spring-run phenotypes have ecological, economic, and cultural importance, and are valuable to commerce and ecosystems for their greater fat content (Cook 2017). They also have had long histories with indigenous peoples, including documented ritualistic management by the Yurok, Karok, Hupa, Shasta, and Tolowa (Swezey and Heizer 1977). Due to reliance on cool, clean water in the summer, spring-run salmonids are particularly vulnerable to anthropogenic effects and have dramatically declined (Thompson *et al.* 2019).

Low genetic divergence (e.g., *F*_ST_ < 0.03) between premature and mature migrants within local rivers was found by multiple studies (Allendorf 1977; Chilcote *et al.* 1980; Waples *et al.* 2004; Arciniega *et al.* 2016). Based on these findings, premature migrant forms did not meet the first criterion for ESU status, sufficient reproductive isolation (Waples and Lindley 2018). However, recent genomic studies by Prince *et al.* (2017) and Thompson (2019) have identified a single locus that has a major effect on the migration phenotype and highlighted the potential for the loss of allelic variation at this locus to have significant ecological consequences, leading to legal action (Hess *et al.* 2016; Prince *et al.* 2017; Micheletti *et al.* 2018; Narum *et al.* 2018; NMFS 2018; Thompson *et al.* 2019). Prince *et al.* (2017) conducted a genome-wide association study that identified a single genetic locus (GREB1L) associated with premature migration. Further phylogenetic analyses suggested that the *GREB1L* alleles determining the premature migrant phenotype arose only once in each species, and subsequently spread through dispersal and positive selection.

**Figure 2.**
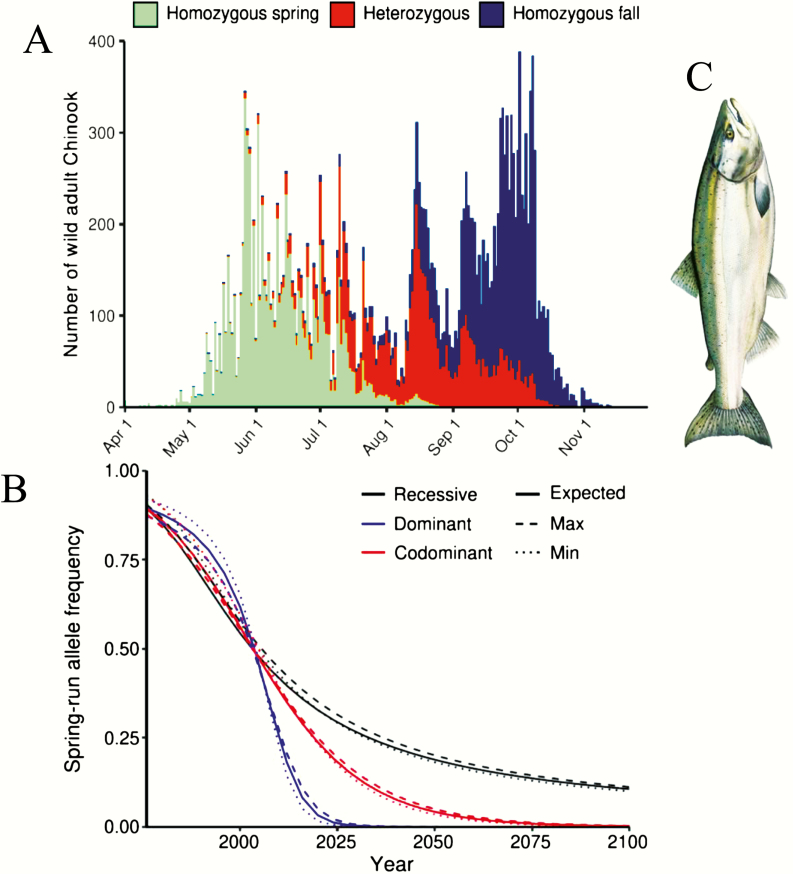
(**A**) Stacked bar graph representing the number of wild adult Chinook salmon passing Gold Ray Fish Counting Station on the Rogue river in 2004; colors represent estimated proportion of each GREB1L locus genotype. (**B**) Selection modeling in Rogue Chinook. Curves representing the decline (or loss) of the spring-run allele frequency over time under a recessive, dominant, or codominant scenario. Spring-run alleles are thought to be codominant and predicted to be lost by ~2075. The modeling assumes random mating and no genetic drift. (**C**) Image of a Chinook salmon. Figure modified from Thompson *et al.* (2019). See online version for full colors.

Thompson *et al.* (2019) further examined selection against the premature migrant phenotype of Chinook salmon in the Rogue River in Oregon after the construction of a dam. They estimated the strength of selection needed to explain the change in allele frequencies at *GREB1L* under multiple dominance scenarios and predicted allele frequencies in future populations. Results suggested that the premature migration allele is probably codominant with respect to fitness and may be lost from the population if the current selection pressure continues (Figure 2B).

Together, these findings suggest that the premature migration phenotype (and allele) is vulnerable to loss and unlikely to reappear for a long time if lost from a population. Populations where *GREB1L* early-migration alleles are prevalent may deserve special legal protection. Based on these results, the Karuk Tribe submitted a petition to list the Klamath premature Chinook under the ESA (NMFS 2018). In February 2018, National Oceanic and Atmospheric Administration (NOAA) Fisheries announced a finding of substantial scientific evidence indicating the creation and listing of a new ESU as threatened or endangered may be warranted. At the time of writing, the National Marine Fisheries status review of the Upper Klamath and Trinity River Chinook salmon was still pending. The decision on whether to list Klamath premature Chinook could have wide-reaching implications for conservation (Waples and Lindley 2018).

These studies and the resultant legal action have recharged debate over whether, when, and how species should be managed for single genes (Kardos and Shafer 2018). A concern is that as genomics continues to make it easier to find adaptive genetic variation, management units could be over-split as more and more important loci and alleles are identified. As this case study in Pacific salmon shows, iterative and focused genomic studies have the power to identify crucial adaptive variation and to inform long-standing debates.

### Effective Population Size and Effective Number of Breeders (*N*_e_ and *N*_b_)

Effective population size (*N*_e_) is one of the most important concepts and parameters in conservation and evolutionary genetics because it influences the rate of loss of genetic variation, the levels of individual inbreeding, and the effectiveness of natural selection and gene flow (Wang *et al.* 2016). Conservation genetics has long employed estimates of effective population size to help assess and monitor the vulnerability of a population to potentially harmful genetic changes as mentioned above.

Although genomic data provide greater resolution and ability to estimate *N*_e_ in a growing diversity of species and scenarios, these data can also present unique challenges in estimating *N*_e_. In his lecture on *N*_e_, Waples discussed the recent advances in theory and computational analysis, which have vastly improved *N*_*e*_ estimation in the genomic era (Waples et al. 2014, 2018a, 2018b; Hollenbeck *et al.* 2016; Zhou *et al.* 2018). The use of thousands of loci, many of which are probably physically linked, will downwardly bias *N*_e_ estimates unless physical location (linkage) is taken into account (Waples and Do 2008; Do *et al.* 2014b; Waples *et al.* 2016).

The recently improved LDNe method implemented in the NeEstimator program (as of version 2.1) improves reliability of confidence intervals and reduces bias in estimating *N*_e_ by calculating *r*^*2*^ on locus pairs, employing positional information from assembled loci or, when available, linkage groups or chromosomes (Do *et al.* 2014a). Likewise, the improved capability of NeEstimator to handle missing data, which calculates a fixed inverse variance-weighted harmonic mean at each locus (Peel *et al.* 2013), has been shown to be accurate with up to 50% missing data (Nunziata and Weisrock 2018). Together, these methodological improvements make estimating effective population size more accessible to studies with reduced representation data (i.e., NGS methods that subsample a genome with a restriction enzyme or targeted capture) with or without a reference genome.

Waples and Andrew Whiteley (University of Montana) highlighted *N*_b_, or the number of effective breeders in a cohort, as a promising parameter for genetic and population management because of its intrinsic relationship to *N*_e_ and potential relationship with population abundance or environmental conditions (Kamath *et al.* 2015). An advantage of estimating *N*_b_, rather than *N*_e_, is that *N*_b_ provides frequent (e.g., yearly) information on population status, rather than having to wait to sample between generations which is often required by temporal estimations of *N*_e_ (e.g., Waples and Yokota 2007; Waples *et al.* 2014).

Whiteley’s lecture emphasized monitoring population cohorts using a single sample and sib-ship or linkage-disequilibrium methods (Kamath *et al.* 2015; Waples *et al.* 2018b) and demonstrated the nuances of estimating *N*_b_ through recent studies of brook trout (*Salvelinus fontinalis*). He cautioned that while estimates of *N*_b_ can track abundance in some species (Ferchaud and Hansen 2016), which may supplement or allow demographic-based monitoring, it is unlikely to closely track abundance for species with high variance in reproductive success and limited reproductive habitat. For example, for brook trout that spawn in available habitat patches *N*_b_ estimates had no association with yearly abundance in 2 populations; however, they provided important information about environmental conditions (Whiteley *et al.* 2015). A comparison among several brook trout populations showed that *N*_b_ was the largest at intermediate flow conditions, which is consistent with biological hypotheses (Whiteley *et al.* 2017).

The theory and application of *N*_b_ was presented mainly in the context of aquatic organisms. Nonetheless, *N*_b_ is easier to estimate than *N*_e_ for most taxa (beyond aquatic organisms), requiring only a single sample per generation (Waples 2005; Waples *et al.* 2013; da Silva *et al.* 2018). Whiteley’s example demonstrated the importance of incorporating detailed biological information in the study design, analysis, and interpretation of effective population size estimates and its relationship to census size (Waples 2005; Waples *et al.* 2013). Simulations, such as those conducted by ConGen 2018 participants with EasyPop (Balloux 2001) and those implemented in tools, such as AGENE (Waples *et al.* 2013), NeOGen (Blower *et al.* 2019), and Neff (Grimm *et al.* 2016), can be employed to determine an appropriate sampling scheme, implement sensitivity analysis, and corroborate empirical results (Waples *et al.* 2013).

## Molecular Genomic Data Generation and Analysis

Training the next generation of conservation genomicists includes empowering participants to evaluate and incorporate a wealth of diverse molecular genetic methods. The first ConGen meetings in 2006–2009 were focused on microsatellites. Since 2010, genomic techniques such as restriction-site associated DNA-sequencing (RADseq) have increasingly been the main focus (Andrews and Luikart 2014). Of 33 participants at ConGen 2018, 27 participants had RADseq data, 4 participants had exon capture data, and 5 participants had whole-genome sequencing (WGS) data. Several participants reported having multiple types of molecular data.

At ConGen 2018, methods both currently applied widely and those only recently employed in conservation genomics were discussed. Paul Hohenlohe (University of Idaho) reviewed the many variations and utility of RADseq (Andrews *et al.* 2016), Stefan Prost (Senckenberg Museum) presented a guide to de novo genome assembly (Fuentes-Pardo and Ruzzante 2017; Hendricks *et al.* 2018), and Rena Schweizer (University of Montana) highlighted the practical and conceptual considerations regarding exon capture (Bi *et al.* 2012; Schweizer *et al.* 2016). Here, we highlight advances in RAD-capture, transcriptomics, and epigenomics.

### Rapture: A Hybrid Reduced Representation Approach

Lectures by Hohenlohe and Seth Smith (University of Montana) demonstrated the utility of Rapture (RAD-capture; Ali *et al.* 2016), a reduced representation technique that combines an improved RADseq library preparation protocol (informally referred to as bestRAD) with an in-solution sequence probe capture to enrich sequencing libraries for a subset of RADseq loci (e.g., polymorphic loci, loci in or near genes, diagnostic loci for species identification or admixture analysis, and/or loci with high heterozygosity or high *F*_ST_, all on the same capture array). The major improvements prescribed by the bestRAD protocol are the ability to reduce the proportion of PCR duplicates, efficiency in using smaller starting quantities of DNA, and efficiency in scaling from hundreds to thousands of samples (Ali *et al.* 2016). We encourage interested readers to see Meek and Larson (2019) for a detailed review of sequence capture techniques and their utility in conservation. Here we focus on the details each individual researcher must weigh in respect to each individual project: cost, PCR duplication rate, and computational approaches.

Because individual (indexed) samples are pooled early in the bestRAD protocol, the cost of the library preparation kit and capture reaction scales well for large sample sizes. For instance, up to 96 uniquely indexed individual samples are pooled prior to adding sequencing adapters and amplifying the library using a commercially available kit. Seth Smith estimated that bestRAD libraries can be generated for *<*$5.00 per individual after the cost of bestRAD adapters is amortized. The per sample cost for the hybridization capture reaction was ~$0.50, assuming the above multiplexing scheme and a bait panel of up to 20 000 loci. This cost could vary substantially depending on the vendor used for supplies (e.g., the capture array) and does not include labor for the data production, which is often the majority of the cost. The cost of sequencing depends on the desired coverage. The number of samples that can be multiplexed per sequencing lane is a function of the number of targeted loci, the PCR duplication rate, and the proportion of reads that do not that align to targeted loci. He cautioned that the PCR duplication rate and proportion of off-target reads are expected to vary depending on the proportion of RAD loci targeted for capture and the total number of loci in the original RAD library which can be influenced by sample quality and PCR duplicate rates, and are typically 20–30% but can be >80% (e.g., Margres *et al.* 2018).

Following sequencing, Rapture data can be analyzed with any method applicable to RAD-type data (Andrews *et al.* 2016). Among these, Stacks (Catchen *et al.* 2013) is commonly used for population genomics with RADseq and has been covered at ConGen since 2011. At ConGen 2018, Amanda Stahlke (University of Idaho) taught de novo and reference-based locus assembly and genotyping in Stacks version 2.3, which has several major changes from the original implementation (Rochette *et al.* 2019). Participants examined the effects on *F*-statistics of removing PCR duplicates and aligning to a reference or not. These choices depend on genetic and financial resources available, local laboratory expertise, and the study question. Useful sensitivity frameworks for assessing RAD locus assembly, the benefits of a reference genome, and the effects of PCR duplication have been described elsewhere (Ebbert *et al.* 2016; Paris *et al.* 2017; Shafer *et al.* 2017; Euclide *et al.* 2020). For example, low-coverage sequencing can be a cost-effective and powerful approach (Maruki and Lynch 2017) but is also the most sensitive to the effects of PCR duplicates (Euclide *et al.* 2020).

As one of the most widely used software pipelines for genotyping RADseq data and population genomic analysis, the Stacks program (Catchen *et al.* 2013) has been discussed and used at the ConGen course for several years. Here we highlight some key changes in the recently released Stacks 2 (Rochette *et al.* 2019) taught at the 2018 course. For users with bestRAD data (Ali *et al.* 2016), the addition of the *--bestrad* flag to process_radtags reorients paired fastq files such that bestRAD indexes and the remainder of restriction cut-sites are always located at the beginning of the first read, eliminating the requirement of an external script to reorient the reads prior to input.

In Stacks 2, users also have the ability to input paired-end reads and assemble local RAD contigs with data produced by protocols with a randomly sheared end (e.g., Ali *et al.* 2016) or random oligos in ddRAD (Schweyen *et al.* 2014). Instead of concatenating forward and reverse reads as previously recommended (Rochette and Catchen 2017), paired-end reads are incorporated through the new tsv2bam and gstacks, the new genotyping module, yielding major improvements in memory usage and genotype-calling frameworks (Rochette *et al.* 2019).

Novel genotype-calling algorithms have also been implemented in gstacks, including the diploid Maruki and Lynch (2017) maximum likelihood genotyping model which can incorporate population-level genotype frequencies (the “low-coverage model”) and error-rates with Bayes’ theorem. In gstacks, users may increase *--alpha* to require a greater statistical threshold for calling genotypes, instead of setting a redundant minimum stacks depth flag in the population module (*-m* is deprecated). These advances in Stacks hold promise to advance RADseq analysis in conservation genomics by yielding more accurate genotypes and longer haplotypes (Rochette *et al.* 2019).

### Transcriptomics and Epigenomics

Transcriptomics and epigenomics, the high-throughput studies of transcribed products and epigenetic modifications of the genome, respectively, can be used to disentangle mechanisms of local adaptation (i.e., plasticity vs. Darwinian adaptation) across biological and temporal scales (Hendricks *et al.* 2018; Kelly 2019), though the application of understanding these mechanisms in conservation is still developing (Christie *et al.* 2016; Le Luyer *et al.* 2017). Recent technological advances in library preparation which better accommodate degraded and low input DNA have made transcriptomic analysis more accessible to systems of conservation concern (Schuierer *et al.* 2017). RNAseq, the high-throughput sequencing of synthesized cDNA fragments (Wang *et al.* 2009), has been used to identify the molecular basis for resilience to changing environment in corals (Barshis *et al.* 2013; Pratlong *et al.* 2015; Bay *et al.* 2017) and redband rainbow trout (*Oncorhynchus mykiss gardieri*; Garvin *et al.* 2015; Chen *et al.* 2018).

Still, there are surprisingly few studies that employ these techniques to inform conservation. Perhaps this is due to fewer labs having the capacity to produce and analyze these potentially tissue- and time-specific data, the actual and perceived conflicts in evolutionary paradigms, or the ongoing discussion regarding the role of plasticity in long-term population persistence (Kelly 2019). Regardless, transgenerational gene expression and epigenetic changes can underlie an adaptive response to environmental change (e.g., corals).

At ConGen 2018, participants gained exposure and experience to transcriptomics through an interactive lecture on data production and hands-on analysis of differential gene expression led by Joanna Kelley (Washington State University). Participants learned how to functionally annotate variants of interest and perform enrichment analysis with instructor Mackenzie Gavery (University of Washington) and an epigenomic data set. Here we highlight Gavery’s lecture demonstrating the potential utility of epigenomics in conservation with a recent study of DNA methylation of cytosine residues at CpG sites induced by hatchery conditions (Gavery et al. 2018, 2019; Box 2).

Box 2. How will changes in DNA methylation influence adaptation to artificial environments in hatchery fish?

A common goal of captive breeding programs is to support declining wild populations (i.e., genetic rescue); however, there is concern that rearing in artificial conditions may inadvertently reduce fitness. In conservation salmonid hatcheries, there is mounting evidence that tank-rearing conditions can induce developmental plasticity and impact life-history traits. To examine the role of epigenetic changes in hatchery-reared steelhead trout (*Oncorhynchus mykiss*), Gavery *et al.* (2019) raised steelhead in an artificial stream and small simulated hatchery tank for 2 years, well past germ cell differentiation, then sampled individuals and performed reduced representation bisulfite sequencing (Meissner *et al.* 2005) to determine methylation patterns. After accounting for familial relationships influencing methylation patterns, they were able to discern up-methylated and down-methylated gene differences between their 2 conditions (artificial stream vs. tank). Although family relatedness had the largest effect, environmental differences also caused significant changes in the methylation pattern. If these epigenetic changes occur at an early stage in development in response to environmental pressures, they may not only affect the organism’s growth, but will continue to persist well past the time when those environmental pressures are no longer present. This has implications for conservation of salmonids and other species if environmentally induced epigenetic shifts are transmitted to offspring and grand offspring. For example, if hatchery-adaptive epigenetic changes are transmitted to wild fish, the fitness of wild fish could decline (Christie *et al.* 2016; Le Luyer *et al.* 2017). There is substantial evidence of maladaptive introgression in wild populations (Rodriguez *et al.* 2019), though more work must be conducted to determine whether epigenetic changes can persist, be transmitted across multiple generations, and spread within and among natural populations (Charlesworth *et al.* 2017).

### Understanding Adaptive Potential and Genomic Vulnerability

Genomic methods now allow researchers to determine the genetic basis for variation in fitness, quantify adaptive capacity, and predict potential outcomes for natural populations facing environmental change (Funk *et al.* 2018). Adaptive potential can be defined as the capacity of species or populations to respond to stressors (e.g., environmental change) by genetically based changes (Nicotra *et al.* 2015; Funk *et al.* 2018). Rachael Bay (University of California Davis) and Christen Bossou (Colorado State University) demonstrated the exciting potential for *genomic vulnerability*, which is an estimate of the extent to which allele frequencies of wild populations must change to maintain current genotype–environment associations in the future (Fitzpatrick and Keller 2015; Box 3).

Box 3. How will genomic vulnerability of yellow warblers influence their evolutionary response to climate change?

**Figure 3.**
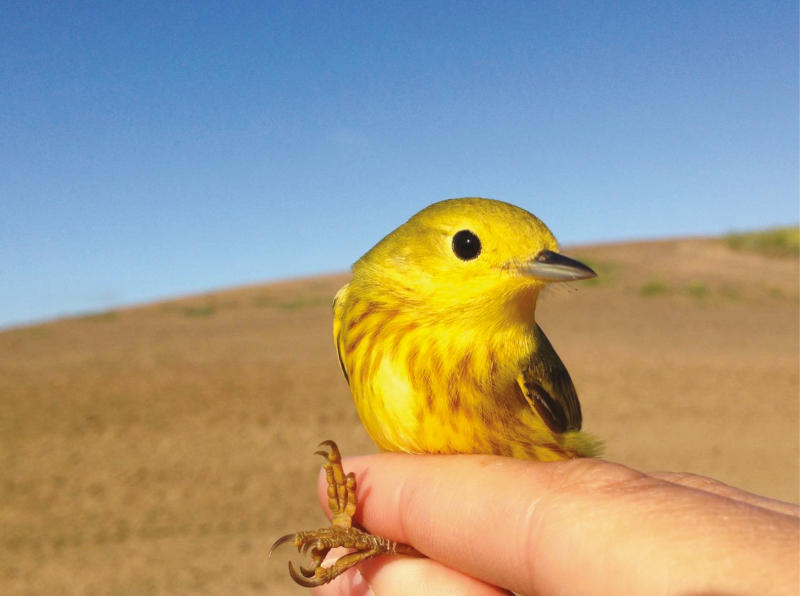
The wide breeding range of the yellow warbler (*Setophaga petechia*), pictured here, and recent population declines in some regions motivated the hands-on tutorial of Bay and Bossou. Photograph by Daniel Karp reproduced with permission.

In their workshop lecture, Bay and Bossou invited participants to assess genomic vulnerability of the yellow warbler (*Setophaga petechia*; Figure 3), a migratory songbird distributed across much of North America (Bay *et al.* 2018). First, participants identified the environmental variables that best explained variation at a subset of genome-wide SNPs using gradient forest analysis, a regression tree-based machine learning approach (Ellis *et al.* 2012). Then, genomic vulnerability was calculated as the difference between current versus predicted gradient forest-transformed climate variables. A significant negative association was found between genomic vulnerability and current population trends, suggesting that populations with high genomic vulnerability may have already been affected (Bay *et al.* 2018). This approach provides a useful starting point to incorporate evolution into models that predict the effects of climate change on biodiversity. Important future extensions of the model could include incorporating additional evolutionary components, such as gene flow and population sizes. Predictive modeling, such as the strategy taught by Bay and Bossou, will become increasingly useful for conservation as it incorporates both local adaptation and projected environmental conditions.

## The Next Generation: Developing Theoretical, Empirical, and Analytical Skills

Conservation genomics is a multidisciplinary field, requiring practitioners to have a working knowledge of population genetic theory and molecular biology while developing the computational skills necessary to apply novel and conventional analyses to increasingly large data sets. These challenges, raised by Allendorf *et al.* (2010), Garner *et al.* (2016), and Shafer *et al.* (2016), remain relevant and were discussed by participants and instructors alike at ConGen 2018. Conservation genomicists often need to navigate social (e.g., legal), ecological, and molecular dimensions, sometimes in the most challenging of field conditions (Groom *et al.* 2006).

Researchers must also be able to effectively communicate with stakeholders, including agency managers, NGOs, policy makers, and the public (Hand *et al.* 2018). The diversity of topics covered during lectures, discussions, and hands-on activities during ConGen 2018 demonstrates the importance of taking a holistic approach when tackling questions in conservation genomics. One recommendation from managers at ConGen to help conservation geneticists ensure their data is used for conservation management was to design a study with a manager who has plans in place (e.g., including permits, policy, etc.) to use the genetic data once it is available to make management decisions (Boyer M, personal communication). This recommendation is an important consideration for future discussion in conservation and genetics workshops where open forums and group conversations can be organized. Other big-group discussion topics ranged from the best programming languages for population genomics (e.g., R and shell scripting), to career choices.

Theory in population genetics has a long and rich history, and yet, is still developing with effective population size concepts and empirical estimation methods among the most important areas (e.g., Waples *et al.* 2014; Ceballos *et al.* 2018; Beaumont and Wang 2019). The importance of theory, and specifically effective population size, is exemplified by the following quotes: “Nothing in evolution makes sense except in light of population genetics” (Lynch and Walsh 2007) and “Nothing in population genetics makes sense except in light of effective population size,” which Robin Waples at ConGen 2018 said was a quote from Fred Allendorf (University of Montana). For example, when testing for genotype–phenotype associations, knowing the effective population size is helpful because *N*_e_ influences the extent of linkage-disequilibrium along chromosomes, which in turn determines the density of markers and molecular methods needed to conduct a powerful genome-wide scan (e.g., Kardos *et al.* 2016).

The increasing diversity and complexity of analysis also requires that code be well annotated and highly reproducible. A number of instructors shared version-controlled worksheets and R code via Github including Racheal Bay, Eric Anderson (Southwest Fisheries Science Center), Joanna Kelley, and Brenna Forester (Colorado State University). Kelley, for example, provided instruction and materials for transcriptome assembly and quantifying differential gene expression (https://github.com/jokelley/congen-2018). Also of discussion was the increasing availability of R packages to efficiently analyze and visualize NGS data sets and results and the importance this has in increasing reproducibility and reliability, and lowering the barrier on bioinformatics and data analysis in general (Paradis *et al.* 2017).

## Summary and Conclusions

In conclusion, major conceptual advances discussed at ConGen 2018 include estimating the effective population size per cohort or generation (e.g., *N*_b_ with age structure, using thousands of loci), assessing population genomic vulnerability, and using adaptive genetic information to identify conservation units. New approaches have emerged for cheaper genome-wide data production (e.g., Rapture) and data analysis (e.g., major updates in Stacks). Emphasis in recent years at ConGen including the use of tools becoming more cost-effective and available to conservation genomics including DNA capture, transcriptomics, epigenomics, genome-wide, and reference-genome-based work. The purpose of ConGen remains to introduce recent novel techniques and approaches to a wide range of participants from different career paths, institutes, and countries. Recent work by ConGen workshop instructors and other researchers has expanded the types of data used in conservation genomics at large (e.g., see transcriptomics and epigenomics and Forester *et al.* 2018). A researcher now often has multiple data types that may include everything from *de novo* genome assemblies to RADseq to differential gene expression among populations and more. Although the amount of genomic data production grows exponentially, the continuing challenge for genomicists remains in obtaining a solid foundation in population genetics theory, data filtering, and computational analysis. Through training and experiences such as those available at workshops like ConGen 2018, the modern conservation and population genomicist will be able to examine a wide range of central questions, evaluate the appropriate tools for data production and analysis, and integrate across different data types from RADseq to whole-genome resequencing, RNAseq, and more. As population genomics continues to evolve, we hope this review of ConGen 2018 will help serve as a benchmark, motivation, and starting point for information and references for readers from world-wide to advance multiple disciplines including conservation, ecology, and evolutionary genomics.
